# In Silico Drug Design and Discovery: Big Data for Small Molecule Design—2nd Edition

**DOI:** 10.3390/biom16040591

**Published:** 2026-04-16

**Authors:** Carmen Cerchia, Antonio Lavecchia

**Affiliations:** Drug Discovery Laboratory, Department of Pharmacy, University of Naples Federico II, 80131 Naples, Italy; antonio.lavecchia@unina.it

The volume and heterogeneity of data available to drug discovery have grown at a pace that would have been difficult to predict even a few years ago. Public repositories of bioactivity measurements, crystallographic coordinates, and gene-expression profiles now collectively catalog billions of data points [1,2,3,4,5,6,7,8,9,10,11,12], while protein structure prediction has opened entire families of targets to computational interrogation [13,14,15,16,17]. At the same time, the way that this data is used has changed. Computational chemistry is no longer a discipline that operates in a silo, feeding one-off predictions into a linear pipeline; instead, it functions as the connective tissue of multidisciplinary discovery campaigns in which machine learning, physics-based simulation, and experimental feedback loops are interwoven from the outset [18,19,20,21,22]. This shift is reshaping the expectations placed on in silico methods [23]. Scale still matters, but it is no longer sufficient on its own. What distinguishes productive computational work today is the quality of the biological question asked, the rigor of the data curation underlying the models, and the willingness to close the loop with experiment. The contributions assembled in this second edition of the *Special Issue* “In Silico Drug Design and Discovery: Big Data for Small Molecule Design” illustrate this maturation across a remarkably diverse set of therapeutic areas and methodological strategies.

A first thematic thread running through the *Special Issue* concerns the use of structural modeling and simulation to illuminate poorly characterized targets. Adewumi and Mosebi tackled estrogen receptor-α36, a clinically relevant but structurally unresolved variant implicated in endocrine-resistant breast cancer [24]. By constructing a homology model [25] of the ligand-binding domain and performing molecular dynamics (MD) simulations coupled with MM/PBSA free-energy calculations, they provided a structural rationale for the preferential binding of broussoflavonol B to ER-α36 over ER-α66, offering a starting point for the design of selective inhibitors. In a different biological context, Giordano et al. combined sequence analysis, phylogenetics, homology modeling, and docking to characterize lipoxygenases from marine diatoms and to propose a classification linked to possible substrate preferences [26]. This study demonstrates how bioinformatic methods can convert sequence data into testable functional hypotheses. Rehman et al. applied a more classical discovery workflow to leucyl-tRNA synthetase from *Mycobacterium tuberculosis*, combining virtual screening with MD and then validating selected hits by isothermal titration calorimetry and antimycobacterial assays [27]. Their identification of Macimorelin as a promising binder of LeuRS underscores how computational repurposing screens, when reinforced by orthogonal experimental evidence, can yield viable chemical leads against drug-resistant pathogens. Yu et al. employed the SILCS methodology and MD to propose that bile acids and their conjugates act as negative allosteric modulators of the M1 muscarinic receptor, with potential implications for colon cancer biology [28]. This work brings together membrane-context signaling, endogenous metabolite pharmacology, and receptor allostery, three dimensions that are often studied in isolation.

A second theme is the transition from single-modality prediction toward integrated, data-rich models that capture multiple dimensions of molecular behavior [29,30]. Umemori et al. addressed a difficult problem in preclinical safety: the prediction of reactive metabolite formation associated with drug-induced liver injury [31,32], by developing a binary classifier for cysteine-trapping outcomes using proprietary experimental data [33]. Their head-to-head comparison of a message-passing neural network and a random forest model is instructive: under a realistic time-split validation, the graph-based architecture outperformed the fingerprint-based model, yet the analysis of contributory substructures retained interpretability in medicinal chemistry terms. This interplay between predictive accuracy and mechanistic transparency reflects a growing expectation in the field. Debnath et al. reported GramSeq-DTA, a framework for drug–target affinity prediction that combines structural representations of compounds and proteins with chemical perturbation signatures derived from the L1000 transcriptomic resource [34]. This study suggests that purely structure-based representations may not capture all the information relevant to affinity prediction. Piazza et al. provide a complementary illustration of how machine learning can be embedded into a practical discovery pipeline [35]. After developing and benchmarking a large panel of predictive models, they used the best performer to drive a virtual screening campaign for CDK9 inhibitors, ultimately identifying compounds with low-micromolar biochemical activity and promising cellular profiles. This work exemplifies how ML can complement rather than replace medicinal chemistry expertise, helping to narrow the candidate pool and focus experimental effort.

A distinct and increasingly important strand of contemporary computational drug design concerns generative models that learn the distribution of chemical space and propose novel molecules rather than simply scoring existing ones [36,37,38,39,40,41]. Islam and Caulfield developed a ligase-conditioned junction tree variational autoencoder to generate synthesizable, drug-like molecular glues aimed at promoting the degradation of amyloid-β42 through the ubiquitin–proteasome system [42]. This contribution is notable because it addresses targeted protein degradation in the context of neurodegeneration and treats generative design as a conditioned problem in which target biology, ligase specificity, and synthetic realism are jointly optimized.

Taken together, the contributions in this *Special Issue* suggest several directions that are likely to define the next phase of big-data-driven drug discovery. First, multimodality is becoming essential [43,44,45]. The most informative models will be those that integrate chemical structure, target structure, transcriptomic perturbation, safety endpoints, and real medicinal chemistry constraints within a single framework, rather than treating each data type separately [46,47]. Second, the coupling of data-driven and physics-based reasoning is gaining ground. Several papers in this *Special Issue* already combine predictive ranking with docking, MD, calorimetry, or cellular phenotyping, and such hybrid strategies are likely to become even more important as the community confronts sparse, noisy, and biased datasets [48]. Third, the standards by which computational work is evaluated are shifting. Retrospective benchmark performance alone is no longer a convincing endpoint; what matters is whether a model can deliver biologically meaningful insight and experimentally testable hypotheses with sufficient robustness [49,50,51]. Alongside these methodological trends, the community would benefit from greater attention to uncertainty quantification, data provenance, negative-result sharing, and model interpretability [52,53,54,55], elements that are still treated as optional refinements.

In conclusion, this second edition of “In Silico Drug Design and Discovery: Big Data for Small Molecule Design” offers a snapshot of a field that is maturing rapidly. The studies collected here indicate that the real promise of big data for small-molecule design is ultimately not automation, but the construction of closed-loop discovery systems in which computation, synthesis, and experiment iteratively refine one another, turning data into decisions, and decisions into better medicines.

## Abbreviations

The following abbreviations are used in this manuscript:

MD	Molecular Dynamics
SILCS	Site identification by ligand competitive saturation
